# Faecal metabolome responses to an altered dietary protein:carbohydrate ratio in adult dogs

**DOI:** 10.1080/01652176.2023.2273891

**Published:** 2023-10-28

**Authors:** Yang Lyu, Jia Xu, Fien Verdoodt, Lynn Vanhaecke, Lieselot Y. Hemeryck, Myriam Hesta

**Affiliations:** a ECAN Equine and Companion Animal Nutrition, Faculty of Veterinary Medicine, Ghent University, Merelbeke, Belgium; b Laboratory of Integrative Metabolomics, Faculty of Veterinary Medicine, Ghent University, Merelbeke, Belgium

**Keywords:** Canine, faecal metabolome, protein intake, dietary carbohydrate, obesity, gut health

## Abstract

High-protein diets may aid weight loss and weight maintenance programs in both humans and dogs, although the effect of dietary protein levels on gut metabolism and functionality has not been studied in depth. The current study aimed to investigate the effect of an altered dietary protein:carbohydrate ratio on gut function in adult dogs by means of faecal metabolomic fingerprinting. More specifically, functional metabolic differences in dogs fed a high-protein/low-carbohydrate (HPLC) *vs.* low-protein/high-carbohydrate (LPHC) diet were studied by equally allocating twelve clinically healthy (6 lean and 6 obese) Beagles into two groups in a cross-over design, with each group receiving two isocaloric diets for four weeks. The faecal metabolome revealed that different protein:carbohydrate ratio can influence host and/or gut microbiome metabolism and function, while no effect was observed on the body condition. Targeted analysis demonstrated that the HPLC diet significantly increased the concentration of indole, spermidine, and pipecolinic acid and decreased the concentration of azelaic acid, D-fructose, mannose, and galactose (*p* < 0.05). Multivariate modelling (OPLS-DA) of the untargeted faecal metabolome revealed distinctly different metabolomic profiles following the HPLC *vs.* LPHC diet, with 18 altered pathways. The HPLC diet influenced amino acid and lipid metabolism, potentially promoting weight loss and immune function, whereas the LPHC diet affected carbohydrate fermentation and may promote anti-oxidative function.

## 1. Introduction

High-protein diets have attracted interest in dogs and humans in recent years, more particularly in the context of weight-loss programs (Weber et al. 2007; German et al. 2010; Andre et al. 2017; Xu et al. 2017). The health effects of high-protein diets are however not fully understood. In dogs, few studies demonstrated that high meat consumption without fibre addition facilitates proteolytic catabolism by the gut microbiota, rather than the more beneficial microbial saccharolytic metabolism (Pinna et al. 2018; Jackson and Jewell 2019). Moreover, dogs fed high-protein diets showed a higher level of faecal pH and specific metabolites, such as branched-chain fatty acids and indole sulphates produced by microbial fermentation of amino acids (Hang et al. 2013; Herstad et al. 2017; Pinna et al. 2018; Jackson and Jewell 2019). The optimum range of protein required for healthy adult dogs has not been determined, neither optimal concentrations nor upper limits recommended (Ephraim et al. 2020). Commercial dog foods contain differing amounts of protein, ranging from 18% to more than 60% (dry matter basis), with equally divergent amino acid concentrations (Ephraim et al. 2020). In humans however, e.g. lower levels of faecal butyrate-producing bacteria and butyrate were observed in obese people consuming a high-protein/low-carbohydrate diet, in association with gut dysbiosis and malfunction (Duncan et al. 2007). Studies in mice also reported that a high-protein/low-carbohydrate diet may have adverse health effects on gut health. Kim et al. (2016) observed similar microbiome changes (e.g. decreased Ruminococcaceae and increased Bacteroides and Parabacteroides) in mouse models of colorectal cancer (Zhu et al. 2014) and colitis (Zackular et al. 2013), as well as in human patients with inflammatory bowel disease (Frank et al. 2007). Since consuming such a diet has become prevalent in dogs’ weight-loss programs (Weber et al. 2007; German et al. 2010; Andre et al. 2017), studies to investigate the health effects of high-protein diets in dogs are therefore also required.

Metabolomics is a holistic approach used to map all small molecules present in a biological matrix, thereby accurately reflecting an individual’s (patho)physiological state (Plekhova et al. 2021). Fecal metabolomics more specifically provides functional readouts of gut microbial metabolism and its interaction with host and environmental factors, including dietary intake (Han et al. 2021). While some canine studies have investigated the impact of high levels of protein on the metabolome and gut health (Hang et al. 2012, 2013; Bermingham et al. 2017), few compared different levels of dietary protein (Herstad et al. 2017; Ephraim et al. 2020), and to date not one study has investigated the effect of high-protein diets in dogs with different body conditions. Using an untargeted high resolution mass spectrometry-based approach, the present study aimed to compare the faecal metabolome of both lean and obese dogs fed a high-protein/low-carbohydrate *vs.* a low-protein/high-carbohydrate diet, to study the influence of dietary protein on gut metabolism. We hypothesized that different dietary protein:carbohydrate ratio influences the formation of faecal metabolites in relation to protein, lipid and/or carbohydrate metabolism, and thereby affects the gut health of dogs. The findings of this study can contribute to better understand potential adverse and/or beneficial health effects of high protein diets in both dogs and humans.

## 2. Materials and methods

### 2.1. Animals and diets

This study was approved by the Ethical Committee of the Faculty of Veterinary Medicine, Ghent University, Belgium (EC 2011/056). Twelve healthy Beagles with a mean age of 6.0 years old were included in this study. Six Beagles (one spayed and three intact females; two intact males) were lean, with a body condition score of 4–5/9, and six Beagles (two intact females and two intact males) were obese, with a body condition score of 8–9/9. Obesity was induced ∼1 year before the present study by feeding the dogs a high-fat commercial diet as described by Van de Velde et al. (2013). Before the study, dogs were deemed healthy (apart from obesity in four dogs), based on physical exams, complete blood counts, and serum biochemistry.

Two isocaloric experimental diets, a high-protein/low-carbohydrate (HPLC) diet consisting of 50.0 g crude protein, 12.2 g ether extract, and 32.2 g nitrogen-free extract on 100 g dry matter basis, and a low-protein/high-carbohydrate (LPHC) diet consisting of 17.8 g crude protein, 13.6 g ether extract, and 62.3 g nitrogen-free extract on 100 g dry matter basis were formulated with the same ingredients (Co. NV Versele-Laga, Deinze, Belgium). Full details of the ingredients and dietary composition were described previously (Xu et al. 2017), and the main ingredients are presented in Supplementary Table S1. Both diets met the minimal requirement for adult dogs according to the National Research Council (2006). The initial amount of feed offered was calculated based on individual maintenance energy requirements according to individual history and adjusted to maintain a stable body weight throughout the study. Dogs were fed twice daily and had free access to water.

The study was designed as a crossover with two 4-week periods. The first 3 weeks consisted of an adaptation period and samples were taken in the fourth week (on day 27). In the first period, three lean and obese dogs were randomly selected and assigned to the LPHC diet first, whilst the other three lean and obese dogs received the HPLC diet. In the second period, diets were switched. Each dog was therefore assigned to one of four groups (group 1: lean dogs received the LPHC diet first; group 2 lean dogs received the HPLC diet first; group 3 obese dogs received the LPHC diet first; group 4 obese dogs received the HPLC diet first). On day 27 of each period, fresh faecal samples were collected within 10 min after spontaneous voiding. An aliquot of ±2 g was placed into a sterile plastic tube, frozen immediately on dry ice, lyophilized as soon as possible, and stored at −80 °C in preparation for metabolomics analysis. Two obese dogs were excluded from the metabolomics analysis due to an insufficient amount of faecal samples, and as such, each group ended up containing three lean and two obese dogs.

### 2.2. Metabolomics

Metabolome extraction, chromatographic separation, and mass spectrometric analysis were performed as previously described using Ultra High-Performance Liquid Chromatography coupled to High-Resolution Mass Spectrometry (UHPLC-HRMS) (Vanden Bussche et al. 2015). Specifically, 200 mg of lyophilized homogenized faeces was mixed with 4 mL of ultrapure water, and 1 mL of a mixture of ice-cold methanol and ultrapure water (80:20) were used for metabolome extraction. Chromatographic separation was performed using an Accela UHPLC system (Thermo Fisher Scientific Inc, Waltham, MA, USA) equipped with an Acquity HSS-T3 C18 column (1.8 μm, 150 × 2.1 mm, Waters) and VanGuard precolumn (1.8 μm, 5 × 2.1 mm, Waters) as reported previously (Vanden Bussche et al. 2015). Mass spectrometric detection was carried out on an Orbitrap high-resolution mass spectrometer (Exactive™, Thermo Fisher Scientific) equipped with a heated electrospray ionization source (HESI-II) (Vanden Bussche et al. 2015).

MS analysis was performed in full scan, enabling targeted and untargeted analysis of the metabolome. For the targeted analysis, a mixture of 120 reference metabolites (detailed in Supplementary Table S2) and internal standard valine-d8 were analysed alongside the samples, under the same instrumental conditions. The reference metabolites were selected based on relevance to the gut metabolome (Vanden Bussche et al. 2015). To assure instrumental precision (mass deviation < 5 ppm), the instrument was calibrated in both polarity modes according to manufacturer recommendations. Pooled quality control (QC) samples, consisting of equal aliquots of all sample extracts, were analysed alongside test specimens at equal intervals to monitor the stability of instrument performance. For the same purpose, the internal standard D-valine-d_8_ was added to all extracted samples at a concentration of 100 ng/mL. The analytical standards (the mixture of 120 metabolites and D-valine-d_8_) were obtained from diverse suppliers, as reported previously (Vanden Bussche et al. 2015).

### 2.3. Data analysis

#### 2.3.1. Targeted analysis

Peak detection and comparison with the reference standard solution mix were performed using Xcalibur™ 3.0 (Thermo Fisher Scientific). Data were normalized by median peak intensity and log transformed before further analysis using the online MetaboAnalyst 5.0 platform (McGill University, Canada). A Student’s *t*-test was carried out to compare the effect of the two diets. To further evaluate the effect of periods and body conditions, two-way ANOVA was performed with diets and periods, and diets and body conditions as factors, respectively. A *p*-value of <0.05 was considered statistically significant and a *p*-value <0.10 was considered a significant trend, after correction using a false discovery rate (FDR; van Iterson et al. 2009).

#### 2.3.2. Untargeted analysis

Untargeted data pre-processing was performed using Sieve™ 2.2 software (Thermo Fisher Scientific), implementing a minimal peak intensity of 500,000 a.u., a maximum peak width of 0.5 min, and a maximum mass deviation of 10 ppm. Multivariate statistical analysis was performed with SIMCA 15.0 (Sartorius, Germany). Data pretreatment included QC normalization, log transformation, and Pareto scaling to achieve approximate normal distribution. Missing values were set to the mean of the two QC neighbouring non-zero values (Kamleh et al. 2012). Principal component analysis (PCA) was used for the exploration of inherent sample clustering and outlier detection. Orthogonal partial least squares discriminant analysis (OPLS-DA) was employed for multivariate modeling to compare the effects of diets (i.e. HPLC *vs.* LPHC) and body condition (i.e. lean *vs.* obese) on the metabolome. Validation of OPLS-DA models was based on the total variation explained by the model (*R*_2_*Y*) and model predictive properties (*Q*_2_) > 0.5, CV-ANOVA *p*-value <0.05, and valid permutation testing (*n* = 100) (Szymańska et al. 2012). For valid models, features contributing the most to group separation were selected based on variable importance in projection (VIP) score >1.0, a correlation |*p*(corrected)| > 0.5, and covariance |*p*| > 0.02 derived from the S-plot, and Jack-knifed confidence intervals not including zero. Tentative annotation was performed by a Human Metabolome Database (HMDB) and Kyoto Encyclopaedia of Genes and Genomes (KEGG) database search, based on accurate *m/z*, with a mass deviation tolerance of 5 ppm. Putative identities were cross-checked with the features’ ^12^C/^13^C isotope ratio, corresponding to Tier level 4 confidence level of identification (Schymanski et al. 2014).

Pathway analysis was performed using the MetaboAnalyst 5.0 platform, applying the ‘Functional Analysis’ module based on the Mummichog algorithm. Pathway significance was determined by enrichment analysis with Holm adjustment for multiple comparisons, based on *m/z*, *p*-values, and statistical scores of all metabolites. The following settings were applied: mass accuracy: 5 ppm; analytical mode: mixed; *p*-value cutoff: 0.05; library: Homo sapiens [MFN] (given the lack of a canine library). Pathway visualization was performed using the MetScape 3.1 App on the Cytoscape 3.5 platform (Institute for Systems Biology, Seattle, WA, USA).

## 3. Results

### 3.1. Targeted metabolomics

**Figure 1.**
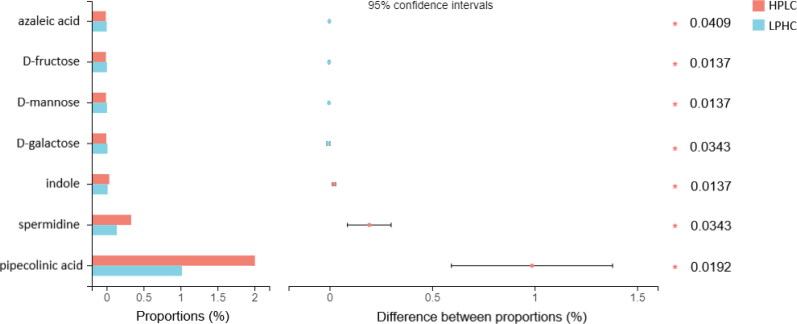
Relative abundance of significantly altered metabolites in dogs fed a low-protein/high carbohydrate (LPHC) *vs.* high-protein/low carbohydrate (HPLC) diet. Bar charts with proportions on the left represent the relative abundance of the average peak intensity of the metabolite in diet groups. Values and ranges of 95% confidence intervals for metabolite relative abundances are presented on the right bars. Asterisks with numbers indicate significant differences and corrected *p*-values.

A total of 73 metabolites were detected in the targeted analysis, of which seven metabolites were significantly different between the two diets (Figure 1). Dogs fed an LPHC diet displayed a higher level of azaleic acid (*p*** **=** **0.0409), D-fructose (*p*** **=** **0.0137), D-mannose (*p*** **=** **0.0137), and D-galactose (*p*** **=** **0.0343); while dogs fed an HPLC diet displayed a higher level of indole (*p*** **=** **0.0137), spermidine (*p* = 0.0343), and pipecolinic acid (*p*** **=** **0.0192). Non-significant findings (FDR corrected *p*** **>** **0.05) are available in Supplementary Table S3. No interaction with the study period and body condition was detected (Supplementary Table S4).

### 3.2. Untargeted metabolomics

A total of 3483 and 1754 ions were obtained in the positive and negative ionization mode, respectively (Supplementary Table S5). PCA-X score plots revealed clustering of faecal samples according to diet (Figure S1), as well as good clustering of QC samples. For dogs fed an LPHC *vs.* HPLC diet, a significant difference in the faecal metabolic fingerprint could be observed, as demonstrated by good descriptive and predictive properties of the OPLS-DA model, i.e. an *R*_2_*Y* of 0.997, *Q*_2_ of 0.793, CV-ANOVA *p*-value of 0.038 and valid permutation test (Supplementary Figure S1). A total of 82 metabolites was retained through VIP, Jack-knifed confidence interval, and S-plot filtering, with 16 unidentified metabolites and 66 putatively annotated metabolites (Supplementary Table S6). Six unidentified and 15 putatively annotated metabolites were defined to be markers of the HPLC diet, whereas 29 unidentified and 32 putatively annotated metabolites were retrieved as markers of the LPHC diet (Supplementary Table S6). Regarding body condition, no valid OPLS-DA model of the faecal metabolic fingerprint in lean *vs.* obese dogs could be obtained.

### 3.3. Pathway analysis

The Mummichog algorithm identified 17 metabolic pathways that were significantly altered by the HPLC and LPHC diets (Supplementary Figure S2), including purine metabolism, N-glycan biosynthesis, vitamin B3 metabolism, hexose phosphorylation, aspartate and asparagine metabolism, linoleate metabolism, 3-oxo-10R-octadecatrienoate beta-oxidation, vitamin B5 biosynthesis, alkaloid biosynthesis, tryptophan metabolism, urea cycle/amino group metabolism, omega-3 fatty acid metabolism, ubiquinone biosynthesis, CoA catabolism, lysine metabolism, tyrosine metabolism, and leukotriene metabolism (*p*** **<** **0.05). In addition, a significant trend was observed for the pyrimidine metabolism pathway (*p*** **<** **0.10).

## 4. Discussion

The use of metabolomics in canine studies is steadily increasing, with a focus on studying the metabolome in dogs with a pathological *vs.* healthy state (Guard et al. 2015; Lawrence et al. 2019; Li et al. 2020). Although a few canine studies have reported differences in metabolomic profiles related to a high intake of dietary protein, these were conducted either for a weight loss program (Bermudez Sanchez et al. 2021) or with a relatively low concentration of protein (Apper et al. 2020). The present study, however, was the first to study and compare the effect of both high and low dietary protein levels (isoenergetic exchange of protein for carbohydrate) on the faecal metabolome of lean and obese dogs.

As indicated by OPLS-DA and pathway analysis, this study revealed distinctly different metabolomic profiles in dogs fed an HPLC *vs.* LPHC diet. These results are in line with expectations, as the micro-ecological environment, i.e. the gut microbiota and its metabolites, are indeed mainly influenced by undigested dietary carbohydrates and proteins (Xu et al. 2017), which is consistent with other recent findings in dogs. In an untargeted metabolomic study by Bermudez Sanchez et al. (2021) for example, a weight loss program based on feeding dogs a high-fibre/high-protein diet also induced a prominent shift in the faecal metabolome, in which levels of 13 compounds were shown to be significantly increased or decreased (Bermudez Sanchez et al. 2021). The faecal metabolome also varied significantly when comparing the metabolome of dogs fed a commercial diet *vs.* dogs fed bones and raw food, which contain a high amount of protein and fat (Schmidt et al. 2018). In a long-term feeding trial with three levels of dietary protein, differences in serum, urine, and faecal metabolites were also observed (Ephraim et al. 2020).

In the current study, a total of 82 metabolites were obtained as marker molecules in the untargeted analysis, with 21 markers of the HPLC diet and 61 markers of the LPHC diet. Thirteen of these putatively annotated metabolites were identified as food components (Supplementary Table S6), whilst the remaining 48 metabolites may be attributed to affected metabolic pathways. In particular, the HPLC diet increased most of the putatively annotated metabolites associated with amino acid and protein metabolism, including amino acids (i.e. 5-hydroxylysine) and di- or tripeptides (e.g. cysteinyl-aspartate, isoleucyl-prolyl-serine, etc.). Besides protein metabolism, lipid metabolism showed significant changes as well, i.e. increased butyryl carnitine following intake of the HPLC diet, and several fatty acid esters (e.g. cervonoyl ethanolamide) and secondary bile acids (e.g. allocholic acid, lithocholic acid) increased following intake of the LPHC diet. Butyryl carnitine is involved in lipid degradation and oxidation (Mels et al. 2011), whilst fatty acid esters are the precursor molecules for lipid biosynthesis (Athenstaedt and Daum 2006). Bile acids regulate lipid metabolism and are known to protect against diet-related obesity (Qi et al. 2015). Total secondary bile acids were also found to negatively correlate with gastrointestinal microbial dysbiosis in dogs with chronic enteropathy (Galler et al. 2022).

**Figure 2.**
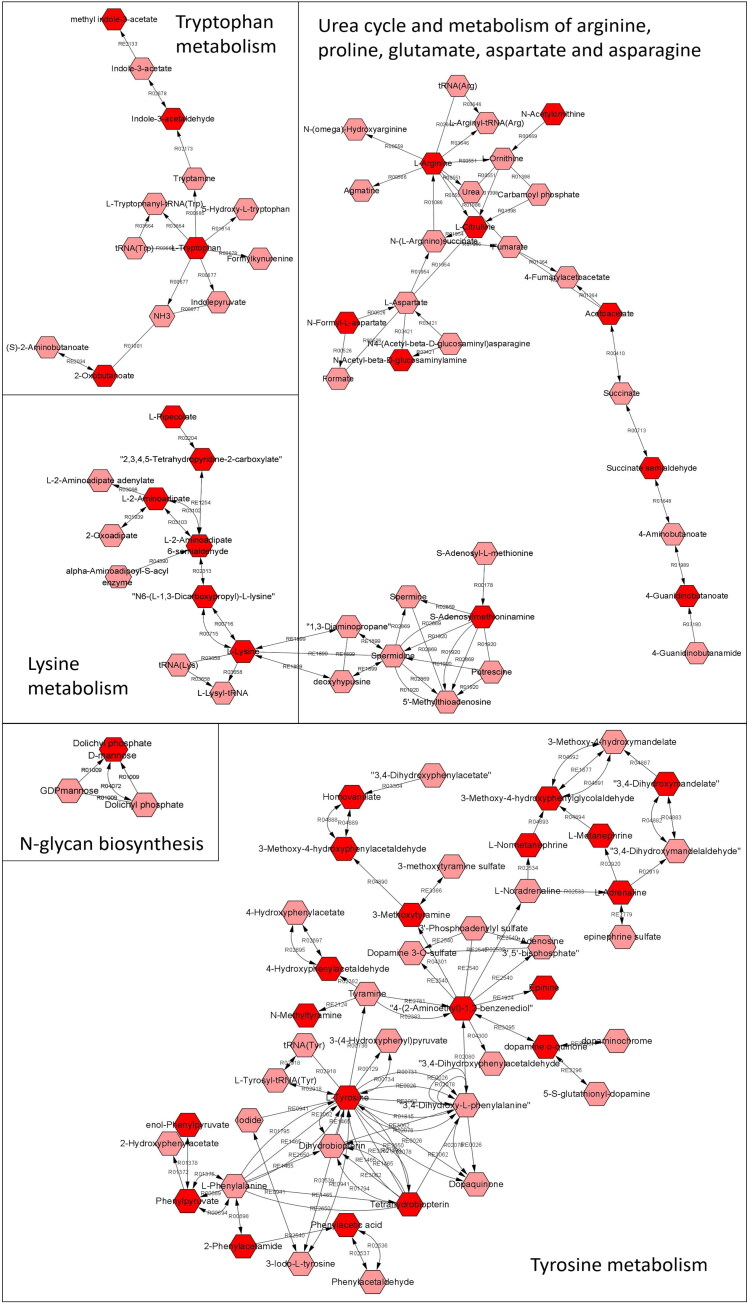
Overview of metabolic pathways associated with amino acid metabolism with significant differences. Red hexagons are compounds (Tier level 5 according to Schymanski et al. 2014) significantly affected by the diets; pink hexagons are detected metabolites linked to the pathway detected, but not significantly different between groups.

**Figure 3.**
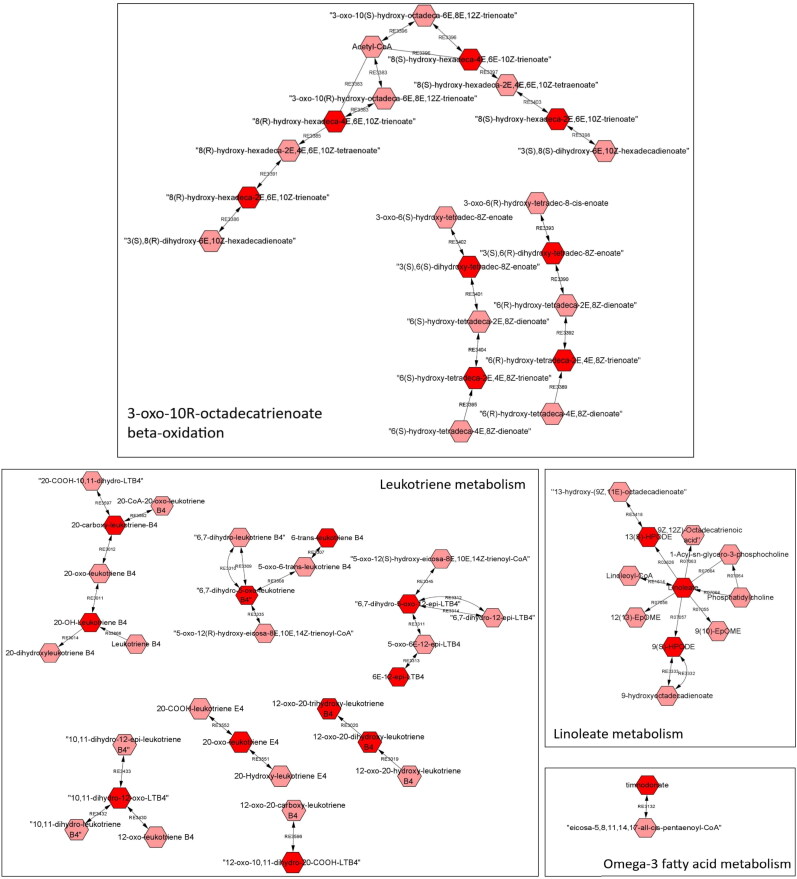
Overview of metabolic pathways associated with fat and fatty acid metabolism with significant differences. Red hexagons are compounds (Tier level 5 according to Schymanski et al. 2014) significantly affected by the diets; pink hexagons are detected metabolites linked to the pathway detected, but not significantly different between groups.

Pathway analysis for the effects of HPLC *vs.* LPHC diet revealed alterations in 18 metabolic pathways. Firstly, seven of these are indeed associated with amino acid metabolism (Figure 2), including aspartate and asparagine, tryptophan, urea cycle/amino group, lysine, and tyrosine metabolism, and N-glycan and alkaloid biosynthesis. Overall, it is not surprising that dietary protein intake alters amino acid metabolism, i.e. not only because proteins consist of amino acids, but also because gut microbial biosynthesis of amino acids increases following increased dietary protein intake (Lin et al. 2017). Secondly, in this study, five fatty acid-related pathways (linoleate, 3-oxo-10R-octadecatrienoate beta-oxidation, omega-3 fatty acid, and leukotriene metabolism and CoA catabolism) were significantly altered by protein:carbohydrate ratio (Figure 3). Other studies have demonstrated that a high-protein/low-fat diet does indeed influence lipid metabolism; for example, by improving the rate of fat loss during a weight loss trial (German et al. 2010) and reducing perirenal adipose tissue in male mice (Wang et al. 2022).

The current work also observed a significant difference in purine and a trend in shifting pyrimidine metabolism. Purines and pyrimidines are essential building blocks for DNA and RNA, for which synthesis is closely regulated (Reaves et al. 2013). Furthermore, purine and pyrimidine metabolism are involved in e.g. cell cycle homeostasis, and immune function (Garavito et al. 2015; Yin et al. 2018). A link between the intestinal microbiome and both purine and pyrimidine degradation and signalling has been demonstrated in humans (Guo et al. 2016; Li et al. 2023), and therefore, it can be hypothesized that a shift in the intestinal microbiota induced by the diet may have caused the alteration in purine and pyrimidine metabolism in this study.

Lastly, hexose phosphorylation, vitamin B3 metabolism, ubiquinone, and vitamin B5 biosynthesis appeared to be altered significantly. Regarding hexose phosphorylation, untargeted findings were consistent with targeted analysis results, where it was shown that levels of the hexoses fructose, galactose, and mannose were higher in dogs fed LPHC diets (see below). Hexose phosphorylation has been linked to microbial carbohydrate fermentation in ruminants, yeasts, and humans (Martin 1996; Rolland et al. 2001; Wolfe 2015) by many specific bacteria, including, e.g. *Escherichia coli* (Wolfe 2015). Altered ubiquinone and vitamin B5 biosynthesis and vitamin B3 metabolism reflect a shift in the functional activity of vitamin biosynthesis by the gut microbiota (Meganathan 2001; Soto-Martin et al. 2020). Although further investigation is required, these functions were likely influenced by the different levels of protein intake, as vitamin B3 is synthesized from tryptophan as a precursor (Makarov et al. 2019), ubiquinone is produced using tyrosine (Bentinger et al. 2010), and vitamin B5 is synthesized from aspartate and a precursor to the amino acid valine (Leonardi and Jackowski 2007).

Using targeted metabolomics, seven metabolites were altered by protein:carbohydrate ratio in the diets. More specifically, the levels of indole, spermidine, and pipecolinic acid were higher in dogs fed an HPLC diet, while azelaic acid, fructose, mannose, and galactose were higher in dogs fed a LPHC diet. Indole, spermidine, and pipecolinic acid are amino acid metabolites, synthesized by the gut microbiome from tryptophan, arginine (or putrescine), and lysine, respectively (He 2006; Matsumoto and Benno 2007; Liu et al. 2020). Increased levels of indole, spermidine, and pipecolinic acid indicate increased amino acid metabolism. It has been shown that indole and its derivatives maintain intestinal homeostasis, and modulate adipogenesis and immune response (Li et al. 2021). This is in line with our untargeted findings and what was reported by another study (Liu et al. 2020), and we also observed a higher indole level in dogs fed a high-protein diet in a previous study (Xu et al. 2017). Notably, the high-protein diet also increased levels of isovalerate and isobutyrate in the previous study (Xu et al. 2017), the disparity between two studies might be due to the different analytical methods (i.e. UPLC *vs.* gas chromatography) and sampling variability. The use of spermidine e.g. showed promising results in diet-induced obese mice, where it improved weight loss and reduced insulin resistance (Ma et al. 2020), and pipecolic acid has also been linked to reduced fatty oxidation and lipid accumulation (Kim et al. 2011). Azelaic acid is a carboxylic acid produced by bacterial degradation of nonanoic acid, a nine-carbon fatty acid (Todea et al. 2021). Interestingly, increased azelaic acid has been reported to possess protective effects against oxidative stress due to its ability to inhibit reactive oxygen species (Jones 2009), as e.g. demonstrated in mice receiving a high-fat diet (Muthulakshmi and Saravanan 2013). Our observation of an increase of azelaic acid levels in dogs fed the LPHC diet in comparison to the HPLC diet may thus be of interest in view of potential anti-oxidative effects. This is in line with our previous work, which indicated that a high-starch diet may exert anti-oxidative effects compared to a high-fat diet (Lyu et al. 2022).

In the present study, both lean and obese dogs were included to evaluate the potential differential impact of dietary protein:carbohydrate ratio in dogs with different body conditions. We were not able to demonstrate that the dogs’ body condition affected the metabolome, as opposed to previous work by Bermudez Sanchez et al. (2021), where clear OPLS-DA separation and 13 significantly changed metabolites could be observed between dogs before and after weight loss for 2–3 months. In the current study, we failed to construct a valid OPLS-DA model according to body condition. This was very likely due to the limited sample size (only 4 obese and 6 lean dogs), and/or related to the stable body weight and condition or the non-occurrence of morbid obesity (unlike the weight-loss study of Bermudez Sanchez et al.), or linked to the increased variability in the microbiota of the obese dogs (as previously reported by Xu et al. 2017).

Apart from the previously mentioned small sample size, the lack of a combination of metabolomic and microbiome analysis is an important limitation. Further research is warranted to increase sample size, and explore metabolome-microbiome correlations and functionality, to further explain and confirm these findings, and how this relates to host metabolism in dogs. Additionally, since metabolomics is emerging in canine nutrition, detailed information about the identification and functionality of many metabolites is often extrapolated from humans or other mammals. Once new, species-specific knowledge becomes available in the future, study interpretations may be updated.

## 5. Conclusion

Based on both targeted and untargeted metabolomic profiling and fingerprinting, the present study observed distinct metabolic differences in dogs fed an HPLC *vs.* an LPHC diet. Targeted analysis revealed seven metabolites significantly altered by the diets, which may be linked to compositional and functional shifts in the microbiome. More specifically, the HPLC diet influenced amino acid and lipid metabolism, potentially promoting weight loss and immunity, whereas the LPHC diet influenced carbohydrate fermentation and may thus promote anti-oxidative function. Pathway analysis revealed that a total of 18 functional pathways were affected, including, amino acid, fatty acid, and nucleic acid metabolism. Future investigations are required to explore the host-microbiome interaction to link the changed metabolome with host metabolism.

## Supplementary Material

Supplemental Material

## Data Availability

The datasets used and/or analysed during the current study are available from the corresponding author upon reasonable request.

## References

[CIT0001] Andre A, Leriche I, Chaix G, Thorin C, Burger M, Nguyen P. 2017. Recovery of insulin sensitivity and optimal body composition after rapid weight loss in obese dogs fed a high-protein medium-carbohydrate diet. J Anim Physiol Anim Nutr. 101 Suppl 1(S1):21–30. doi: 10.1111/jpn.12744. 28627053

[CIT0002] Apper E, Privet L, Taminiau B, Le Bourgot C, Svilar L, Martin JC, Diez M. 2020. Relationships between gut microbiota, metabolome, body weight, and glucose homeostasis of obese dogs fed with diets differing in prebiotic and protein content. Microorganisms. 8(4):513. doi: 10.3390/microorganisms8040513. 32260190 PMC7232476

[CIT0003] Athenstaedt K, Daum G. 2006. The life cycle of neutral lipids: synthesis, storage and degradation. Cell Mol Life Sci. 63(12):1355–1369. doi: 10.1007/s00018-006-6016-8. 16649142 PMC11136409

[CIT0004] Bentinger M, Tekle M, Dallner G. 2010. Coenzyme Q–biosynthesis and functions. Biochem Biophys Res Commun. 396(1):74–79. doi: 10.1016/j.bbrc.2010.02.147. 20494114

[CIT0005] Bermingham EN, Maclean P, Thomas DG, Cave NJ, Young W. 2017. Key bacterial families (Clostridiaceae, Erysipelotri­chaceae and Bacteroidaceae) are related to the digestion of protein and energy in dogs. PeerJ. 5:e3019. doi: 10.7717/peerj.3019. 28265505 PMC5337088

[CIT0006] Bermudez Sanchez S, Pilla R, Sarawichitr B, Gramenzi A, Marsilio F, Steiner JM, Lidbury JA, Woods GRT, Suchodolski JS, German AJ, et al. 2021. Untargeted fecal metabolome analysis in obese dogs after weight loss achieved by feeding a high-fiber-high-protein diet. Metabolomics. 17(7):66. doi: 10.1007/s11306-021-01815-1. 34228201 PMC8260550

[CIT0007] Duncan SH, Belenguer A, Holtrop G, Johnstone AM, Flint HJ, Lobley GE. 2007. Reduced dietary intake of carbohydrates by obese subjects results in decreased concentrations of butyrate and butyrate-producing bacteria in feces. Appl Environ Microbiol. 73(4):1073–1078. doi: 10.1128/AEM.02340-06. 17189447 PMC1828662

[CIT0009] Ephraim E, Cochrane CY, Jewell DE. 2020. Varying protein levels influence metabolomics and the gut microbiome in healthy adult dogs. Toxins. 12(8):517. doi: 10.3390/toxins12080517. 32806674 PMC7472411

[CIT0010] Frank DN, St. Amand AL, Feldman RA, Boedeker EC, Harpaz N, Pace NR. 2007. Molecular-phylogenetic characterization of microbial community imbalances in human inflammatory bowel diseases. Proc Natl Acad Sci USA. 104(34):13780–13785. doi: 10.1073/pnas.0706625104. 17699621 PMC1959459

[CIT0011] Galler AI, Suchodolski JS, Steiner JM, Sung CH, Hittmair KM, Richter B, Burgener IA. 2022. Microbial dysbiosis and fecal metabolomic perturbations in Yorkshire Terriers with chronic enteropathy. Sci Rep. 12(1):12977. doi: 10.1038/s41598-022-17244-6. 35902689 PMC9334271

[CIT0012] Garavito MF, Narváez-Ortiz HY, Zimmermann BH. 2015. Pyrimidine metabolism: dynamic and versatile pathways in pathogens and cellular development. J Genet Genomics. 42(5):195–205. doi: 10.1016/j.jgg.2015.04.004. 26059768

[CIT0013] German AJ, Holden SL, Bissot T, Morris PJ, Biourge V. 2010. A high protein high fibre diet improves weight loss in obese dogs. Vet J. 183(3):294–297. doi: 10.1016/j.tvjl.2008.12.004. 19138868

[CIT0014] Guard BC, Barr JW, Reddivari L, Klemashevich C, Jayaraman A, Steiner JM, Vanamala J, Suchodolski JS. 2015. Characterization of microbial dysbiosis and metabolomic changes in dogs with acute diarrhea. PLOS One. 10(5):e0127259. doi: 10.1371/journal.pone.0127259. 26000959 PMC4441376

[CIT0015] Guo Z, Zhang J, Wang Z, Ang KY, Huang S, Hou Q, Su X, Qiao J, Zheng Y, Wang L, et al. 2016. Intestinal microbiota distinguish gout patients from healthy humans. Sci Rep. 6:20602. doi: 10.1038/srep20602. 26852926 PMC4757479

[CIT0016] Han S, Van Treuren W, Fischer CR, Merrill BD, DeFelice BC, Sanchez JM, Higginbottom SK, Guthrie L, Fall LA, Dodd D, et al. 2021. A metabolomics pipeline for the mechanistic interrogation of the gut microbiome. Nature. 595(7867):415–420. doi: 10.1038/s41586-021-03707-9. 34262212 PMC8939302

[CIT0017] Hang I, Heilmann RM, Grützner N, Suchodolski JS, Steiner JM, Atroshi F, Sankari S, Kettunen A, de Vos WM, Zentek J, et al. 2013. Impact of diets with a high content of greaves-meal protein or carbohydrates on faecal characteristics, volatile fatty acids and faecal calprotectin concentrations in healthy dogs. BMC Vet Res. 9(1):201. doi: 10.1186/1746-6148-9-201. 24107268 PMC3851871

[CIT0018] Hang I, Rinttila T, Zentek J, Kettunen A, Alaja S, Apajalahti J, Harmoinen J, de Vos WM, Spillmann T. 2012. Effect of high contents of dietary animal-derived protein or carbohydrates on canine faecal microbiota. BMC Vet Res. 8(1):90. doi: 10.1186/1746-6148-8-90. 22735212 PMC3464166

[CIT0019] He M. 2006. Pipecolic acid in microbes: biosynthetic routes and enzymes. J Ind Microbiol Biotechnol. 33(6):401–407. doi: 10.1007/s10295-006-0078-3. 16418868

[CIT0020] Herstad KMV, Gajardo K, Bakke AM, Moe L, Ludvigsen J, Rudi K, Rud I, Sekelja M, Skancke E. 2017. A diet change from dry food to beef induces reversible changes on the faecal microbiota in healthy, adult client-owned dogs. BMC Vet Res. 13(1):147. doi: 10.1186/s12917-017-1073-9. 28558792 PMC5450340

[CIT0021] Jackson MI, Jewell DE. 2019. Balance of saccharolysis and proteolysis underpins improvements in stool quality induced by adding a fiber bundle containing bound polyphenols to either hydrolyzed meat or grain-rich foods. Gut Microbes. 10(3):298–320. doi: 10.1080/19490976.2018.1526580. 30376392 PMC6546335

[CIT0022] Jones DA. 2009. Rosacea, reactive oxygen species, and azelaic acid. J Clin Aesthet Dermatol. 2:26. PMC2958186 20967185

[CIT0023] Kamleh MA, Ebbels TM, Spagou K, Masson P, Want EJ. 2012. Optimizing the use of quality control samples for signal drift correction in large-scale urine metabolic profiling studies. Anal Chem. 84(6):2670–2677. doi: 10.1021/ac202733q. 22264131

[CIT0024] Kim E, Kim DB, Park JY. 2016. Changes of mouse gut microbiota diversity and composition by modulating dietary protein and carbohydrate contents: a pilot study. Prev Nutr Food Sci. 21(1):57–61. doi: 10.3746/pnf.2016.21.1.57. 27069907 PMC4827636

[CIT0025] Kim H-J, Kim JH, Noh S, Hur HJ, Sung MJ, Hwang J-T, Park JH, Yang HJ, Kim M-S, Kwon DY, et al. 2011. Metabolomic analysis of livers and serum from high-fat diet induced obese mice. J Proteome Res. 10(2):722–731. doi: 10.1021/pr100892r. 21047143

[CIT0026] Lawrence YA, Bishop MA, Honneffer JB, Cook AK, Rodrigues-Hoffmann A, Steiner JM, Suchodolski JS, Lidbury JA. 2019. Untargeted metabolomic profiling of serum from dogs with chronic hepatic disease. J Vet Intern Med. 33(3):1344–1352. doi: 10.1111/jvim.15479. 30891842 PMC6524095

[CIT0027] Leonardi R, Jackowski S. 2007. Biosynthesis of pantothenic acid and coenzyme A. EcoSal Plus. 2(2):11–28. doi: 10.1128/ecosalplus.3.6.3.4. PMC4950986 26443589

[CIT0028] Li M, Liu B, Li R, Yang P, Leng P, Huang Y. 2023. Exploration of the link between gut microbiota and purinergic signalling. Purinergic Signal. 19(1):315–327. doi: 10.1007/s11302-022-09891-1. 36121551 PMC9984663

[CIT0029] Li Q, Laflamme DP, Bauer JE. 2020. Serum untargeted metabolomic changes in response to diet intervention in dogs with preclinical myxomatous mitral valve disease. PLOS One. 15(6):e0234404. doi: 10.1371/journal.pone.0234404. 32555688 PMC7302913

[CIT0030] Li X, Zhang B, Hu Y, Zhao Y. 2021. New insights into gut-bacteria-derived indole and its derivatives in intestinal and liver diseases. Front Pharmacol. 12:769501. doi: 10.3389/fphar.2021.769501. 34966278 PMC8710772

[CIT0031] Lin R, Liu W, Piao M, Zhu H. 2017. A review of the relationship between the gut microbiota and amino acid metabolism. Amino Acids. 49(12):2083–2090. doi: 10.1007/s00726-017-2493-3. 28932911

[CIT0032] Liu Y, Hou Y, Wang G, Zheng X, Hao H. 2020. Gut microbial metabolites of aromatic amino acids as signals in host–microbe interplay. Trends Endocrinol Metab. 31(11):818–834. doi: 10.1016/j.tem.2020.02.012. 32284282

[CIT0033] Lyu Y, Liu D, Nguyen P, Peters I, Heilmann RM, Fievez V, Hemeryck LY, Hesta M. 2022. Differences in metabolic profiles of healthy dogs fed a high-fat *vs.* a high-starch diet. Front Vet Sci. 9:801863. doi: 10.3389/fvets.2022.801863. 35252418 PMC8891928

[CIT0034] Ma L, Ni Y, Wang Z, Tu W, Ni L, Zhuge F, Zheng A, Hu L, Zhao Y, Zheng L, et al. 2020. Spermidine improves gut barrier integrity and gut microbiota function in diet-induced obese mice. Gut Microbes. 12(1):1–19. doi: 10.1080/19490976.2020.1832857. PMC7668533 33151120

[CIT0035] Makarov MV, Trammell SA, Migaud ME. 2019. The chemistry of the vitamin B3 metabolome. Biochem Soc Trans. 47(1):131–147. doi: 10.1042/BST20180420. 30559273 PMC6411094

[CIT0036] Martin SA. 1996. Hexose phosphorylation by the ruminal bacterium *Selenomonas ruminantium*. J Dairy Sci. 79(4):550–556. doi: 10.3168/jds.S0022-0302(96)76399-3. 8744219

[CIT0037] Matsumoto M, Benno Y. 2007. The relationship between microbiota and polyamine concentration in the human intestine: a pilot study. Microbiol Immunol. 51(1):25–35. doi: 10.1111/j.1348-0421.2007.tb03887.x. 17237596

[CIT0038] Meganathan R. 2001. Ubiquinone biosynthesis in microorganisms. FEMS Microbiol Lett. 203(2):131–139. doi: 10.1111/j.1574-6968.2001.tb10831.x. 11583838

[CIT0039] Mels C, Jansen van Rensburg P, van der Westhuizen FH, Pretorius PJ, Erasmus E. 2011. Increased excretion of c4-carnitine species after a therapeutic acetylsalicylic acid dose: evidence for an inhibitory effect on short-chain fatty acid metabolism. ISRN Pharmacol. 2011:851870. doi: 10.5402/2011/851870. 22084721 PMC3199914

[CIT0040] Muthulakshmi S, Saravanan R. 2013. Protective effects of azelaic acid against high-fat diet-induced oxidative stress in liver, kidney and heart of C57BL/6J mice. Mol Cell Biochem. 377(1–2):23–33. doi: 10.1007/s11010-013-1566-1. 23361364

[CIT0041] National Research Council. 2006. Energy. In: Nutrient requirements of dogs and cats. Washington (DC): National Academies; p. 28–48.

[CIT0042] Pinna C, Vecchiato CG, Bolduan C, Grandi M, Stefanelli C, Windisch W, Zaghini G, Biagi G. 2018. Influence of dietary protein and fructooligosaccharides on fecal fermentative end-products, fecal bacterial populations and apparent total tract digestibility in dogs. BMC Vet Res. 14(1):106. doi: 10.1186/s12917-018-1436-x. 29558912 PMC5859515

[CIT0043] Plekhova V, De Paepe E, Van Renterghem K, Van Winckel M, Hemeryck LY, Vanhaecke L. 2021. Disparities in the gut metabolome of post-operative Hirschsprung’s disease patients. Sci Rep. 11(1):16167. doi: 10.1038/s41598-021-95589-0. 34373532 PMC8352975

[CIT0044] Qi Y, Jiang C, Cheng J, Krausz KW, Li T, Ferrell JM, Gonzalez FJ, Chiang JYL. 2015. Bile acid signaling in lipid metabolism: metabolomic and lipidomic analysis of lipid and bile acid markers linked to anti-obesity and anti-diabetes in mice. Biochim Biophys Acta. 1851(1):19–29. doi: 10.1016/j.bbalip.2014.04.008. 24796972 PMC4219936

[CIT0045] Reaves ML, Young BD, Hosios AM, Xu YF, Rabinowitz JD. 2013. Pyrimidine homeostasis is accomplished by directed overflow metabolism. Nature. 500(7461):237–241. doi: 10.1038/nature12445. 23903661 PMC4470420

[CIT0046] Rolland F, Wanke V, Cauwenberg L, Ma P, Boles E, Vanoni M, et al. 2001. The role of hexose transport and phosphorylation in cAMP signalling in the yeast Saccharomyces cerevisiae. FEMS Yeast Res. 1:33–45. 12702461 10.1111/j.1567-1364.2001.tb00011.x

[CIT0047] Schmidt M, Unterer S, Suchodolski JS, Honneffer JB, Guard BC, Lidbury JA, Steiner JM, Fritz J, Kölle P. 2018. The fecal microbiome and metabolome differs between dogs fed bones and raw food (BARF) diets and dogs fed commercial diets. PLOS One. 13(8):e0201279. doi: 10.1371/journal.pone.0201279. 30110340 PMC6093636

[CIT0048] Schymanski EL, Jeon J, Gulde R, Fenner K, Ruff M, Singer HP, Hollender J. 2014. Identifying small molecules via high resolution mass spectrometry: communicating confidence. Environ Sci Technol. 48(4):2097–2098. doi: 10.1021/es5002105. 24476540

[CIT0049] Soto-Martin EC, Warnke I, Farquharson FM, Christodoulou M, Horgan G, Derrien M, Faurie J-M, Flint HJ, Duncan SH, Louis P, et al. 2020. Vitamin biosynthesis by human gut butyrate-producing bacteria and cross-feeding in synthetic microbial communities. mBio. 11(4):e00886-20. doi: 10.1128/mBio.00886-20. 32665271 PMC7360928

[CIT0050] Szymańska E, Saccenti E, Smilde AK, Westerhuis JA. 2012. Double-check: validation of diagnostic statistics for PLS-DA models in metabolomics studies. Metabolomics. 8(Suppl 1):3–16. doi: 10.1007/s11306-011-0330-3. 22593721 PMC3337399

[CIT0051] Todea A, Deganutti C, Spennato M, Asaro F, Zingone G, Milizia T, Gardossi L. 2021. Azelaic acid: a bio-based building block for biodegradable polymers. Polymers. 13(23):4091. doi: 10.3390/polym13234091. 34883592 PMC8659112

[CIT583417] Van De Velde H, Janssens GPJ, Rochus K, Duchateau L, Scharek-Tedin L, Zentek J, Nguyen P, Cox E, Buyse J, Biourge V, et al. 2013. Proliferation capacity of T-lymphocytes is affected transiently after a long-term weight gain in Beagle dogs. Vet Immunol Immunopathol. 152(3-4):237–244. 10.1016/j.vetimm.2012.12.011. 23333192 23333192

[CIT0052] van Iterson M, ‘t Hoen PA, Pedotti P, Hooiveld GJ, den Dunnen JT, van Ommen GJ, Boer JM, Menezes RX. 2009. Relative power and sample size analysis on gene expression profiling data. BMC Genom. 10:439. 10.1186/1471-2164-10-439 PMC2759969 19758461

[CIT0053] Vanden Bussche J, Marzorati M, Laukens D, Vanhaecke L. 2015. Validated high resolution mass spectrometry-based approach for metabolomic fingerprinting of the human gut phenotype. Anal Chem. 87(21):10927–10934. doi: 10.1021/acs.analchem.5b02688. 26451617

[CIT0054] Wang K, Peng X, Yang A, Huang Y, Tan Y, Qian Y, Lv F, Si H. 2022. Effects of diets with different protein levels on lipid metabolism and gut microbes in the host of different genders. Front Nutr. 9:940217. doi: 10.3389/fnut.2022.940217. 35782952 PMC9240812

[CIT0055] Weber M, Bissot T, Servet E, Sergheraert R, Biourge V, German AJ. 2007. A high‐protein, high‐fiber diet designed for weight loss improves satiety in dogs. J Vet Intern Med. 21(6):1203–1208. doi: 10.1111/j.1939-1676.2007.tb01939.x. 18196727

[CIT0056] Wolfe AJ. 2015. Glycolysis for microbiome generation. Microbiol Spectr. 3(3):3. doi: 10.1128/microbiolspec.MBP-0014-2014. PMC4507297 26185089

[CIT0057] Xu J, Verbrugghe A, Lourenço M, Cools A, Liu DJX, Van de Wiele T, Marzorati M, Eeckhaut V, Van Immerseel F, Vanhaecke L, et al. 2017. The response of canine faecal microbiota to increased dietary protein is influenced by body condition. BMC Vet Res. 13(1):374. doi: 10.1186/s12917-017-1276-0. 29202841 PMC5716228

[CIT0058] Yin J, Ren W, Huang X, Deng J, Li T, Yin Y. 2018. Potential mechanisms connecting purine metabolism and cancer therapy. Front Immunol. 9:1697. doi: 10.3389/fimmu.2018.01697. 30105018 PMC6077182

[CIT0059] Zackular JP, Baxter NT, Iverson KD, Sadler WD, Petrosino JF, Chen GY, Schloss PD. 2013. The gut microbiome modulates colon tumorigenesis. mBio. 4(6):e00692-13. doi: 10.1128/mBio.00692-13. 24194538 PMC3892781

[CIT0060] Zhu Q, Jin Z, Wu W, Gao R, Guo B, Gao Z, Yang Y, Qin H. 2014. Analysis of the intestinal lumen microbiota in an animal model of colorectal cancer. PLOS One. 9(6):e90849. doi: 10.1371/journal.pone.0090849. 24603888 PMC3946251

