# Supplementary material for: Faecal metabolome responses to an altered dietary protein:carbohydrate ratio in adult dogs
Source: Vet Q. 2023 Oct 28;43(1):1–10. doi: 10.1080/01652176.2023.2273891 (PMC10614716; doi:10.1080/01652176.2023.2273891)
Supplement: Supplemental Material [file TVEQ_A_2273891_SM0737.zip › Supplementary data/Supplementary_Material_Oct_15clean.doc]

**Table S1.** Main ingredients of experimental diets.

| **Ingredients (g/100 g)** | **LPHC** | **HPLC** |
| --- | --- | --- |
| Pork greaves | 11 | 53.3 |
| Brewers rice | 50.9 | 20 |
| Lard | 11 | 8.5 |
| Rice meal | 15 | 6 |
| Beet pulp | 3.7 | 5 |
| Dicalcium phosphate | 3 | 2.1 |
| Yeast | 1 | 1 |
| Salmon Oil | 1 | 1 |
| Animal digest^*^ | 0.89 | 0.89 |
| Calcium carbonate | 0.4 | 0.5 |
| Bentonite clay | 0.5 | 0.5 |
| Salt | 0.81 | 0.42 |
| Vitamin mix | 0.26 | 0.23 |
| Mineral mix | 0.22 | 0.22 |
| Chorine chloride | 0.14 | 0.14 |
| Lecithine | 0.1 | 0.1 |

^*^ Animal digest: a material which results from chemical and/or enzymatic hydrolysis of clean and undecomposed animal tissue.

**Table S2.** Compiled targeted metabolite database: metabolites relevant to gut health (according to Vanden Bussche et al., 2015).

| **N°** | **Compound Name** | **Elemental Composition** | **N°** | **Compound Name** | **Elemental Composition** |
| --- | --- | --- | --- | --- | --- |
| 1 | 1,3-propanediole | C_3_H_8_O_2_ | 61 | Inositol | C_6_H_12_O_6_ |
| 2 | 3-(4-hydroxphenyl)propionic acid | C_9_H_10_O_3_ | 62 | Isobutyric acid | C_4_H_8_O_2_ |
| 3 | 3-phenylpropionic acid | C_9_H_10_O_2_ | 63 | Isovaleric acid | C_5_H_10_O_2_ |
| 4 | 3-hydroxybenzoic acid | C_7_H_6_O_3_ | 64 | L-alanine | C_3_H_7_NO_2_ |
| 5 | 3-hydroxybutyric acid | C_4_H_7_O_3_ | 65 | L-asparaginic acid | C_4_H_7_NO_4_ |
| 6 | 3-hydroxypyridine | C_5_H_5_NO | 66 | L-phenylalanine | C_9_H_11_NO_2_ |
| 7 | 3-Methylhexanedioic acid | C_7_H_12_O_4_ | 67 | L-glutamic acid | C_5_H_9_NO_4_ |
| 8 | 3,4-dihydroxyphenylacetic acid | C_8_H_8_O_4_ | 68 | L-isoleucine | C_6_H_13_NO_2_ |
| 9 | 3,4-dihydroxyphenylpropionic acid | C_9_H_10_O_4_ | 69 | L-leucine | C_6_H_13_NO_2_ |
| 10 | 4-aminobutyric acid | C_4_H_9_NO_2_ | 70 | L-lysine | C_6_H_14_N_2_O_2_ |
| 11 | 4-hydroxy-2-nonenal | C_9_H_16_O_2_ | 71 | L-methionine | C_5_H_11_NO_2_S |
| 12 | 4-hydroxyphenylacetic acid | C_8_H_8_O_3_ | 72 | L-ornithine | C_5_H_12_N_2_O_2_H |
| 13 | 4-methylvaleric acid | C_6_H_12_O_2_ | 73 | L-proline | C_5_H_9_NO_2_ |
| 14 | 5-aminovaleric acid | C_5_H_11_NO_2_ | 74 | L-pyroglutamic acid | C_5_H_7_NO_3_ |
| 15 | 5-methyl-2,4-dihydroxypyrimidine | C_5_H_6_N_2_O_2_ | 75 | L-serine | C_3_H_7_NO_3_ |
| 16 | Adipinic acid | C_6_H_10_O_4_ | 76 | L-threonine | C_4_H_9_NO_3_ |
| 17 | Azelaic acid | C_9_H_16_O_4_ | 77 | L-tyrosine | C_9_H_11_NO_3_ |
| 18 | Acetic acid | C_2_H_4_O_2_ | 78 | L-tryptophan | C_11_H_12_N_2_O_2_ |
| 19 | Benzoic acid | C_7_H_6_O_2_ | 79 | L-valine | C_5_H_11_NO_2_ |
| 20 | Beta-alanine | C_3_H_7_NO_2_ | 80 | Lactic acid | C_3_H_6_O_3_ |
| 21 | Butyric acid | C_4_H_8_O_2_ | 81 | Litocholic acid | C_24_H_40_O_3_ |
| 22 | Cholic acid | C_24_H_40_O_5_ | 82 | Linoleic acid | C_17_H_32_O_2_ |
| 23 | Cadaverine | C_5_H_14_N_2_ | 83 | Malate | C_4_H_6_O_5_ |
| 24 | Caffeic acid | C_9_H_8_O_4_ | 84 | Maleic acid | C_4_H_4_O_4_ |
| 25 | Chenodeoxycholic acid | C_24_H_40_O_4_ | 85 | Malondialdehyde | C_3_H_4_O_2_ |
| 26 | Cholesterol | C_27_H_46_O | 86 | Maltose | C_12_H_22_O_11_ |
| 27 | Citrazinic acid | C_6_H_5_NO_4_ | 87 | Mannose | C_6_H_12_O_6_ |
| 28 | Cortisone | C_21_H_28_O_5_ | 88 | Meso-erythrol (1,2,3,4-tetrahydroxybutane) | C_3_H_10_O_4_ |
| 29 | Cortisol | C_21_H_30_O_5_ | 89 | Methylsuccinate | C_5_H_8_O_4_ |
| 30 | Crotonaldehyde | C_4_H_6_O | 90 | N-Nitroso-N-methylurea | C_2_H_5_N_3_O_2_ |
| 31 | Cyclohexanocarboxylic acid | C_7_H_12_O_2_ | 91 | N-acetylglucosamine | C_8_H_15_NO_6_ |
| 32 | Cysteine | C_3_H_7_NO_2_S | 92 | N-Nitrosodiethylamine | C_4_H_10_N_2_O |
| 33 | Cytosine | C_4_H_5_N_3_O | 93 | N-Nitrosodimethylamine | C_2_H_6_N_2_O |
| 34 | D-Arabinose | C_5_H_10_O_5_ | 94 | Niacine | C_6_H_5_NO_2_ |
| 35 | D-Ribose | C_5_H_10_O_5_ | 95 | Nonanal | C_9_H_18_O |
| 36 | D-fructose | C_6_H_12_O_6_ | 96 | N-piperidine | C_5_H_10_N_2_O |
| 37 | D-valine-d8 | C_5_H_11_O_2_N | 97 | N-Nitrosopyrrolidine | C_4_H_8_N_2_O |
| 38 | D-quinic acid | C_7_H_12_O_6_ | 98 | Octanal | C_8_H_16_O |
| 39 | Dodecanedioic acid | C_12_H_22_O_4_ | 99 | Octanoic acid | C_8_H_16_O_2_ |
| 40 | Dopamine | C_8_H_12_O_2_Cl | 100 | Oleic acid | C_18_H_34_O_2_ |
| 41 | Ethanolamine | C_2_H_7_NO | 101 | p-cresol | C_7_H_8_O |
| 42 | Phenol | C_6_H_6_O | 102 | Palmitic acid | C_16_H_32_O_2_ |
| 43 | Phenylacetic acid | C_8_H_8_O_2_ | 103 | Panthothenic acid | C_9_H_17_NO_5_ |
| 44 | Fumaraat | C_4_H_4_O_4_ | 104 | Pentanal | C_5_H_10_O |
| 45 | Galactose | C_6_H_12_O_6_ | 105 | Pipecolinic acid | C_6_H_11_NO_2_ |
| 46 | Glucaric acid (Saccharic acid) | C_6_H_9_KO_8_ | 106 | Propionic acid | C_3_H_6_O_2_ |
| 47 | Glucitol (Sorbitol) | C_6_H_14_O_6_ | 107 | Putrescine | C_4_H_12_N_2_ |
| 48 | Gluconic acid sodium salt | C_6_H_11_NaO_7_ | 108 | Pyrogallol | C_6_H_6_O_3_ |
| 49 | Glucose | C_6_H_12_O_6_ | 109 | Sarcosine | C_3_H_7_NO_2_ |
| 50 | Glucuronic acid | C_6_H_10_O_7_ | 110 | Sebacic acid | C_10_H_18_O_4_ |
| 51 | Glutaric acid | C_5_H_8_O_4_ | 111 | Spermidine | C_7_H_19_N_3_ |
| 52 | Glycerol | C_3_H_8_O_3_ | 112 | Spermine | C_10_H_26_N_4_ |
| 53 | Glycocholate | C_26_H_43_NO_6_ | 113 | Stearic acid | C_18_H_36_O_2_ |
| 54 | Glycolic acid | C_2_H_4_O_3_ | 114 | Suberic acid | C_8_H_14_O_4_ |
| 55 | Glycine | C_2_H_5_NO_2_ | 115 | Succinate | C_4_H_6_O_4_ |
| 56 | Heptanal | C_7_H_14_O | 116 | Thiazolidine-4-carboxylic acid | C_4_H_7_NO_2_S |
| 57 | Hexanoic acid | C_6_H_12_O_2_ | 117 | Tauroursodeoxycholic acid | C_26_H_45_NO_6_S |
| 58 | Hexanal | C_6_H_12_O | 118 | Ursodeoxycholic acid | C_24_H_40_O_4_ |
| 59 | Indol | C_8_H_7_N | 119 | Valeric acid | C_5_H_10_O_2_ |
| 60 | Indole-3-acetic acid | C_10_H_9_NO_2_ | 120 | Xanthine | C_5_H_4_N_4_O_2_ |

**Table S3.** Non-significant findings of targeted metabolite intensities.

| **Metabolites** | **HPLC** | | **LPHC** | | ***p*-values** | |
| --- | --- | --- | --- | --- | --- | --- |
|  | Mean | SD | Mean | SD | uncorrected | FDR |
| 3-Hydroxybenzoic acid | 132754.9506 | 47424.41079 | 187705.6694 | 48157.7919 | 0.019236 | > 0.05 |
| 3-Hydroxypyridine | 20368.32201 | 6135.987681 | 28354.16551 | 17563.10374 | 0.191427 | > 0.05 |
| 3-Methyladipic acid | 29422.84485 | 6237.873996 | 36497.62331 | 14172.35037 | 0.165688 | > 0.05 |
| 3-Phenylpropionic acid | 8927.516049 | 3678.685909 | 12968.82136 | 16098.38767 | 0.449034 | > 0.05 |
| 3,4-(Dihydroxyphenyl)propionic acid | 267.6872546 | 137.1399068 | 1007.287736 | 1243.311786 | 0.349615 | > 0.05 |
| 3,4-(Hydroxyphenyl)propionic acid | 7322.078958 | 4008.155393 | 22780.45803 | 26559.42434 | 0.085441 | > 0.05 |
| 4-Aminobutyric acid | 4848314.95 | 2708567.254 | 7450319.888 | 5098108.803 | 0.171178 | > 0.05 |
| 4-Hydroxyphenylacetic acid | 100612.8128 | 90333.67667 | 85297.77145 | 63220.80688 | 0.665710 | > 0.05 |
| 4-Methylvaleric acid | 25153.3979 | 12730.00587 | 24218.13036 | 11521.98608 | 0.865161 | > 0.05 |
| 5-Aminovaleric acid | 72056695.09 | 19802009.52 | 78875228.91 | 20016022.74 | 0.453716 | > 0.05 |
| Acetic acid | 1361925.459 | 354303.2507 | 1437466.227 | 330558.4186 | 0.627984 | > 0.05 |
| B-alanine | 14759103.85 | 3367172.525 | 16459817.63 | 4242784.555 | 0.333917 | > 0.05 |
| Benzoic acid | 34097.19622 | 8323.745268 | 44783.53942 | 18484.90047 | 0.112831 | > 0.05 |
| Butyric acid | 361891.8658 | 62804.7517 | 465235.9231 | 138037.248 | 0.044956 | > 0.05 |
| Cadaverine | 3153140.607 | 3121382.291 | 1946104.16 | 2273894.304 | 0.336062 | > 0.05 |
| Caffeic acid | 13460.82827 | 8295.028003 | 18726.84737 | 11348.83561 | 0.251568 | > 0.05 |
| Chenodeoxycholic acid | 171717.5082 | 145368.9893 | 163440.8151 | 94512.16184 | 0.881695 | > 0.05 |
| Cholic acid | 3714220.98 | 4814504.856 | 4176429.423 | 5328701.037 | 0.841007 | > 0.05 |
| Cyclohexenecarboxylicacid | 47676.9144 | 18061.80633 | 54252.76334 | 18926.84327 | 0.437062 | > 0.05 |
| D-arabinose | 60854.90974 | 25484.43907 | 113699.0814 | 48424.211 | 0.006835 | > 0.05 |
| Dodecanedioic acid | 494234.4384 | 509816.6923 | 598746.9564 | 768159.4089 | 0.724155 | > 0.05 |
| Ethanolamine | 96330.40812 | 20297.24418 | 68564.1876 | 24063.43075 | 0.012115 | > 0.05 |
| Fumaric acid | 2159394.235 | 1112242.594 | 2726146.129 | 1503935.648 | 0.350688 | > 0.05 |
| Glucose | 916971.129 | 450854.5418 | 1520951.299 | 811132.3852 | 0.054357 | > 0.05 |
| Glucuronic acid | 910693.9955 | 449156.3857 | 1471895.419 | 793634.719 | 0.067427 | > 0.05 |
| Glutaric acid | 7854.692563 | 5437.968886 | 21638.29607 | 26463.80741 | 0.124061 | > 0.05 |
| Glycerol | 7789.939892 | 2848.341937 | 21418.33345 | 26060.58258 | 0.277519 | > 0.05 |
| Glycine | 1884207.78 | 440797.214 | 1997371.327 | 700724.0667 | 0.670675 | > 0.05 |
| Hexanoic acid | 25868.25507 | 12439.70317 | 24205.56012 | 11683.628 | 0.761551 | > 0.05 |
| Indole-3-acetic acid | 152269.7544 | 105427.6727 | 336931.7151 | 266523.5151 | 0.056588 | > 0.05 |
| Isobutyric acid | 1896325.247 | 323793.3462 | 2367282.799 | 833899.0483 | 0.113253 | > 0.05 |
| Isovaleric acid | 284048.8092 | 241362.1088 | 237724.2079 | 236537.9249 | 0.669818 | > 0.05 |
| L-alanine | 15949538.49 | 2599817.714 | 16724237.42 | 4247647.737 | 0.628722 | > 0.05 |
| L-aspartic acid | 3530498.545 | 1269799.182 | 4307224.97 | 1264475.547 | 0.187334 | > 0.05 |
| L-glutamic acid | 19032667.65 | 10484224.03 | 28874945.91 | 17446174.5 | 0.143616 | > 0.05 |
| L-isoleucine | 127053936.2 | 31895799.31 | 148539656.3 | 47604538.23 | 0.251153 | > 0.05 |
| L-lysine | 11085218.88 | 3615223.44 | 10988377.11 | 5318410.632 | 0.962543 | > 0.05 |
| L-methionine | 15395797.23 | 4740866.579 | 16076191.88 | 4951367.049 | 0.757228 | > 0.05 |
| L-phenylalanine | 78018532.81 | 22971503.43 | 77630794.89 | 27421562.89 | 0.973034 | > 0.05 |
| L-proline | 19977816.24 | 5760192.309 | 18626058.39 | 5575412.701 | 0.600400 | > 0.05 |
| L-pyroglutamic acid | 1440568.928 | 333342.9978 | 1448464.12 | 599057.8012 | 0.971350 | > 0.05 |
| L-serine | 2423653.219 | 937094.7199 | 2920440.606 | 1020304.847 | 0.271671 | > 0.05 |
| L-threonine | 4173693.718 | 1499649.692 | 5580889.6 | 2390300.331 | 0.132209 | > 0.05 |
| L-tyrosine | 4852528.62 | 1693897.171 | 6141235.944 | 2197061.709 | 0.159103 | > 0.05 |
| L-valine | 108939375.5 | 20155911.06 | 93453412.81 | 24891290.28 | 0.143655 | > 0.05 |
| Maleic acid | 2092012.58 | 1164558.618 | 2546917.428 | 1408735.519 | 0.441496 | > 0.05 |
| Malic acid | 5233980.465 | 2623747.797 | 3967336.949 | 2602194.396 | 0.292702 | > 0.05 |
| Methylsuccinic acid | 58143.66363 | 31539.2423 | 76546.85546 | 65013.44838 | 0.435558 | > 0.05 |
| N-acetylglucosamine | 482325.9571 | 415175.5166 | 1343579.923 | 2239920.84 | 0.247390 | > 0.05 |
| Nicotinic acid | 3403650.65 | 1474666.618 | 4847376.816 | 1177804.847 | 0.026373 | > 0.05 |
| N-nitrosamine | 645927.7124 | 295699.5761 | 653914.9296 | 228108.5598 | 0.956367 | > 0.05 |
| N-nitrosopyrrolidine | 290378.5719 | 106846.8035 | 309606.4719 | 80838.33761 | 0.655380 | > 0.05 |
| Octanoic acid | 19633.92278 | 6329.24381 | 19302.58063 | 9106.882046 | 0.92577 | > 0.05 |
| Pantothenic acid | 782330.5576 | 373295.4393 | 1106348.569 | 590937.3733 | 0.159920 | > 0.05 |
| Phenylacetic acid | 301404.6585 | 47504.04075 | 409685.4034 | 111825.0589 | 0.011381 | > 0.05 |
| Propionic acid | 3254488.933 | 938593.7195 | 3033278.176 | 1332818.604 | 0.672927 | > 0.05 |
| Putrescine | 1803120.718 | 1060297.961 | 2158652.741 | 1185960.411 | 0.48878 | > 0.05 |
| Sarcosine | 15684565.42 | 2553714.811 | 16471101.54 | 4258123.194 | 0.622486 | > 0.05 |
| Sebacic acid | 126590.334 | 31816.91555 | 110077.5098 | 45672.04618 | 0.360596 | > 0.05 |
| Spermine | 177523.0109 | 107263.8888 | 132536.6677 | 74814.16596 | 0.291036 | > 0.05 |
| Suberic acid | 130592.0949 | 19180.02595 | 135369.1437 | 42069.6412 | 0.747644 | > 0.05 |
| Succinic acid | 643226.4893 | 1094905.056 | 203889.5369 | 311012.9058 | 0.327224 | > 0.05 |
| Thiazolidine-4-carboxylic acid | 34234.25611 | 29762.16974 | 41502.3164 | 18592.78704 | 0.520785 | > 0.05 |
| Ursodeoxycholic acid | 2330702.996 | 1950396.67 | 3030741.034 | 1762438.088 | 0.410769 | > 0.05 |
| Valeric acid | 875001.4257 | 253522.9943 | 520799.3063 | 211728.7737 | 0.003255 | > 0.05 |

**Table S4.** Summary of t-test *p*-values for the comparison between diet and period, and between diet and body condition (BC).

| ***p*-values (FDR)** | **Diet vs. Period** | | | **Diet vs. BC** | | |
| --- | --- | --- | --- | --- | --- | --- |
|  | **Diet** | **Period** | **Interaction** | **Diet** | **BC** | **Interaction** |
| Indole | 0.0122 | 0.684 | 0.7944 | 0.0224 | 0.7940 | 0.722 |
| mannose | 0.0122 | 0.9318 | 0.6314 | 0.0224 | 0.6753 | 0.979 |
| Pipecolinic acid | 0.0122 | 0.5472 | 0.798 | 0.0304 | 0.8633 | 0.709 |
| D-fructose | 0.0122 | 0.9318 | 0.6314 | 0.0224 | 0.7940 | 0.526 |
| galactose | 0.0456 | 0.9318 | 0.6314 | 0.0304 | 0.5195 | 0.623 |

**Table S5.** Overview of retrieved features using the untargeted metabolomics approach.

Provided as excel file ‘Supplementary Table S5’ for positive (sheet 1) and negative (sheet 2) ionisation separately. QC: quality control; L: lean; O: obese; P: period.

**Table S6.** Summary of the retrieved putatively annotated (Tier level 4 according to Schymanski et al., 2014)) untargeted discriminative metabolites (RT: retention time (min)).

| **Group** | **Compounds** | **Formula** | ***m/z*** | **RT** | **Ion mode** | **Δppm** | **Database** |
| --- | --- | --- | --- | --- | --- | --- | --- |
| HPLC | Isoleucyl-prolyl-serine | C_14_H_25_N_3_O_5_ | 315.16602 | 1.44 | + | 1.448 | PubChem |
| HPLC | Nb-Methyltetrahydroharmol | C_13_H_16_N_2_O | 216.15984 | 9.53 | + | 7.055 | HMDB |
| HPLC | 2'',6''-o-diacetyloninin | C_9_H_12_N_4_O_2_ | 208.09737 | 8.38 | + | 0.783 | HMDB |
| HPLC | (3-Aminopropoxy)guanidine | C_4_H_12_N_4_O | 132.10219 | 7.80 | + | 6.146 | HMDB |
| HPLC | Betaine aldehyde hydrate | C_5_H_14_NO_2_ | 120.08105 | 8.38 | + | 6.135 | HMDB |
| HPLC | Cysteinyl-Aspartate/Aspartyl-Cysteine | C_7_H_12_N_2_O_5_S | 235.12401 | 2.95 | + | 3.269 | HMDB |
| HPLC | Rhamnitol/Fucitol | C_6_H_14_O_5_ | 166.08658 | 8.38 | + | 2.940 | HMDB |
| HPLC | 5-Hydroxylysine | C_6_H_14_N_2_O_3_ | 162.09169 | 8.38 | + | 8.129 | HMDB |
| HPLC | Butyrylcarnitine | C_11_H_21_NO_4_ | 231.17096 | 7.61 | + | 0.146 | HMDB |
| HPLC | PA(8:0/12:0) | C_23_H_45_O_8_P | 480.30799 | 9.42 | + | 12.920 | HMDB |
| HPLC | MG(5-iso PGF2VI/0:0/0:0) | C_21_H_36_O_7_ | 399.18765 | 4.47 | + | 9.735 | HMDB |
| HPLC | Pro-Thr-Pro/Pro-Pro-Thr/Thr-Pro-Pro | C_14_H_23_N_3_O_5_ | 313.18756 | 6.67 | + | 0.572 | PubChem |
| HPLC | 2-Nonanone | C_9_H_18_O | 142.05037 | 4.21 | - | 4.200 | PubChem |
| HPLC | Thr-His-Val/Val-Thr-His/Val-His-Thr/Ser-Ile-His/Ser-His-Leu/Leu-His-Ser/… | C_15_H_25_N_5_O_5_ | 355.19925 | 5.53 | - | 6.422 | PubChem |
| HPLC | Leupeptin | C_20_H_38_N_6_O_4_ | 426.23605 | 6.69 | - | 5.756 | HMDB |
| LPHC | Mesobilirubinogen | C_33_H_44_N_4_O_6_ | 591.31726 | 9.07 | + | 0.144 | HMDB |
| LPHC | Lithocholate | C_24_H_39_O_3_ | 375.28900 | 11.37 | + | 1.284 | KEGG |
| LPHC | Cervonoyl ethanolamide | C_24_H_36_O_3_ | 373.27362 | 11.39 | + | 1.113 | HMDB |
| LPHC | Dehydropipernonaline | C_21_H_25_NO_3_ | 339.26810 | 11.37 | + | 1.391 | HMDB |
| LPHC | 8-Hydroxy-5,6-octadienoic acid | C_8_H_12_O_3_ | 156.07687 | 0.89 | + | 13.390 | HMDB |
| LPHC | 3-Mercaptohexyl butyrate | C_10_H_20_O_2_S | 204.08672 | 1.87 | + | 3.502 | HMDB |
| LPHC | (Allo)cholic acid | C_24_H_40_O_5_ | 408.31070 | 11.39 | + | 1.407 | HMDB |
| LPHC | Glu-Ala-Asn-Lys-Glu | C_23_H_39_N_7_O_11_ | 589.30206 | 9.41 | + | 0.415 | PubChem |
| LPHC | PA(8:0/12:0) | C_23_H_45_O_8_P | 408.31070 | 10.85 | + | 12.920 | HMDB |
| LPHC | Kanzonol I | C_27_H_32_O_5_ | 436.34171 | 11.37 | + | 2.233 | HMDB |
| LPHC | N,N-Dimethylaniline N-oxide | C_8_H_11_NO | 137.04604 | 3.09 | + | 12.550 | HMDB |
| LPHC | Dehydropipernonaline | C_21_H_25_NO_3_ | 339.26852 | 10.73 | + | 1.391 | HMDB |
| LPHC | Mesobilirubinogen | C_33_H_44_N_4_O_6_ | 591.31818 | 9.34 | + | 0.144 | HMDB |
| LPHC | Dehydropipernonaline | C_21_H_25_NO_3_ | 339.26837 | 11.62 | + | 1.391 | HMDB |
| LPHC | Ser-His-Val-Lys | C_20_H_35_N_7_O_6_ | 468.25003 | 8.92 | + | 6.142 | PubChem |
| LPHC | 5a-Cholestane-3a,7a,12a,23,25-pentol | C_27_H_48_O_5_ | 452.33682 | 11.37 | + | 10.670 | HMDB |
| LPHC | Lysyl-proline | C_11_H_21_N_3_O_3_ | 243.21068 | 11.37 | + | 0.729 | HMDB |
| LPHC | Trp-His-Phe/Phe-His-Trp/His-Phe-Trp/Try-Phe-His | C_26_H_28_N_6_O_4_ | 488.24558 | 11.36 | + | 8.610 | PubChem |
| LPHC | Dehydropipernonaline | C_21_H_25_NO_3_ | 339.26813 | 11.99 | + | 1.391 | HMDB |
| LPHC | De-O-methylsimmondsin | C_15_H_23_NO_9_ | 361.29077 | 11.37 | + | 3.204 | HMDB |
| LPHC | N-Nonanoylglycine | C_11_H_21_NO_3_ | 215.17947 | 11.37 | + | 2.141 | HMDB |
| LPHC | 3a,7a,12a-Trihydroxy-5b-cholestan-26-al/3alpha,7alpha-Dihydroxycoprostanic acid/3alpha,7alpha,24(S)-trihydroxy-5beta-cholestan-27-al | C_27_H_46_O_4_ | 434.32642 | 10.82 | + | 0.898 | HMDB |
| LPHC | Valyl-Methionine/Methionyl-Valine | C_10_H_20_N_2_O_3_S | 248.12921 | 11.37 | + | 1.158 | HMDB |
| LPHC | [(1R,2S,4R,5S,6S,10S,11R,12R,14R,15R)-5,6,11,15-Tetrahydroxy-4-(hydroxymethyl)-8,12-dimethyl-7-oxo-14-prop-1-en-2-yl-3-oxatetracyclo[9.4.0.02,4.06,10]pentadec-8-en-14-yl] (2E,4E)-pentadeca-2,4-dienoate | C_35_H_52_O_9_ | 617.40704 | 11.37 | + | 12.760 | PubChem |
| LPHC | (Allo)cholic acid | C_24_H_40_O_5_ | 408.31073 | 11.66 | + | 1.407 | HMDB |
| LPHC | Lovastatin acid/Monoketocholic acid | C_24_H_38_O_6_ | 422.32623 | 11.37 | + | 0.381 | HMDB |
| LPHC | N-oleoyl aspartic acid/N-oleoyl-L-glutamic acid/12-(Methacryloylcarbamoyloxy)stearic acid | C_22_H_39_NO_5_ | 391.28583 | 10.74 | - | 6.146 | PubChem |
| LPHC | N-oleoyl aspartic acid/N-oleoyl-L-glutamic acid/12-(Methacryloylcarbamoyloxy)stearic acid | C_22_H_39_NO_5_ | 391.28577 | 11.64 | - | 0.697 | PubChem |
| LPHC | N-oleoyl aspartic acid/N-oleoyl-L-glutamic acid/12-(Methacryloylcarbamoyloxy)stearic acid | C_22_H_39_NO_5_ | 391.28577 | 11.97 | - | 0.889 | PubChem |
| LPHC | N-oleoyl aspartic acid/N-oleoyl-L-glutamic acid/12-(Methacryloylcarbamoyloxy)stearic acid | C_22_H_39_NO_5_ | 391.28586 | 10.48 | - | 0.873 | PubChem |
| LPHC | 3-Methoxytyrosine | C_10_H_13_NO_4_ | 211.06110 | 9.11 | - | 1.352 | HMDB |
| LPHC | Leu-Glu-Glu/Glu-Leu-Glu/Glu-Glu-Leu | C_16_H_27_N_3_O_8_ | 389.27005 | 10.31 | - | 0.457 | PubChem |
| HPLC | Unidentified | - | 86.09691 | 7.80 | + | - | - |
| HPLC | Unidentified | - | 100.07613 | 5.05 | + | - | - |
| HPLC | Unidentified | - | 100.07620 | 5.35 | + | - | - |
| HPLC | Unidentified | - | 100.07603 | 4.45 | + | - | - |
| HPLC | Unidentified | - | 100.07602 | 4.80 | + | - | - |
| HPLC | Unidentified | - | 428.25101 | 6.67 | + | - | - |
| LPHC | Unidentified | - | 415.28159 | 11.36 | + | - | - |
| LPHC | Unidentified | - | 437.29114 | 10.67 | - | - | - |
| LPHC | Unidentified | - | 427.26215 | 11.36 | - | - | - |
| LPHC | Unidentified | - | 437.29080 | 11.64 | - | - | - |
| LPHC | Unidentified | - | 437.29086 | 11.96 | - | - | - |
| LPHC | Unidentified | - | 785.59283 | 10.75 | + | - | - |
| LPHC | Unidentified | - | 490.24554 | 11.36 | + | - | - |
| LPHC | Unidentified | - | 785.59314 | 11.62 | + | - | - |
| LPHC | Unidentified | - | 798.58844 | 10.85 | + | - | - |
| LPHC | Unidentified | - | 785.59198 | 11.37 | + | - | - |
| LPHC | Unidentified | - | 154.06168 | 0.84 | - | - | - |
| LPHC | Unidentified | - | 413.26620 | 10.82 | + |  |  |
| LPHC | Unidentified | - | 377.29599 | 10.75 | + | - | - |
| LPHC | Unidentified | - | 357.27853 | 11.37 | + | - | - |
| LPHC | Unidentified | - | 357.27887 | 10.73 | + | - | - |
| LPHC | Unidentified | - | 391.28519 | 12.24 | - | - | - |
| LPHC | Unidentified | - | 391.28574 | 11.37 | - | - | - |
| LPHC | Unidentified | - | 357.10193 | 9.19 | - | - | - |
| LPHC | Unidentified | - | 375.28918 | 11.62 | + | - | - |
| LPHC | Unidentified | - | 357.27884 | 10.99 | + | - | - |
| LPHC | Unidentified | - | 375.28918 | 11.96 | + | - | - |
| LPHC | Unidentified | - | 375.28943 | 10.49 | + | - | - |
| LPHC | Unidentified | - | 357.27872 | 12.25 | + | - | - |
| LPHC | Unidentified | - | 357.27875 | 11.62 | + | - | - |
| LPHC | Unidentified | - | 357.27869 | 12.00 | + | - | - |
| LPHC | Unidentified | - | 410.32617 | 11.37 | + | - | - |
| LPHC | Unidentified | - | 410.32651 | 11.62 | + | - | - |
| LPHC | Unidentified | - | 410.32648 | 11.96 | + | - | - |
| LPHC | Unidentified | - | 321.25766 | 11.37 | + | - | - |

**Figure S1**. OPLS-DA score plot of the LPHC vs. HPLC faecal metabolome (R^2^Y = 0.997, Q^2^ = 0.793, CV-ANOVA *p*-value = 0.038, good permutation testing).

**Figure S2.** A total of 17 metabolic pathways were significantly affected, and 1 pathway demonstrated a trend with a *p* < 0.10 (marked in purple), when comparing the faecal metabolome in dogs fed HPLC vs. LPHC. Bars represent the negative log_10_ of the *p*-value and the ratio between brackets represents the number of significant matches to the total number of matched compounds in the pathway.
